# Multidimensional diversity of birds in different habitats of the wetland complex in Huaibei Plain, eastern China

**DOI:** 10.3897/BDJ.13.e154264

**Published:** 2025-05-29

**Authors:** Yongmin Li, Huidong Xu, Xiaoyu Wang, Xu Yong, Dongwei Li, Yatao Wu, Wenfeng Hu

**Affiliations:** 1 Fuyang Normal University, Fuyang, China Fuyang Normal University Fuyang China

**Keywords:** wetland, habitat, bird, multidimensional, diversity

## Abstract

Wetlands are amongst the most productive ecosystems around the globe, with lots of ecological and social-economic services provided. Better understanding of the spatial distribution of biotic communities in wetlands is not only an important question in ecology, but also critical for biodiversity conservation. From March 2022 to February 2023, 12 monthly surveys were conducted on 10 provincially significant wetlands in the Huaibei Plain using transect and point-count methods. In this study, a two-way ANOVA was used to compare the differences in species, taxonomic, functional and phylogenetic diversity of birds across the four seasons and the six habitats. A total of 129,916 individuals, 218 bird species belonging to 54 families and 18 orders were identified during the survey. During spring, species richness was significantly higher in rivers (55.67 ± 4.93), shelterbelts (47.67 ± 6.51) and gardens (44.67 ± 5.29) compared to farmlands, lakes and ponds. In contrast, farmland in summer exhibited greater species richness (SR) (46.67 ± 7.00), Shannon-Wiener (3.13 ± 0.10), Pielou index (0.82 ± 0.02) and phylogenetic diversity (PD) (1431.31 ± 129.92) than shelterbelts, gardens and lakes. River, lake and pond habitats showed notably higher taxonomic diversity (TD), functional diversity (FD) and phylogenetic diversity (PD) during autumn, while the highest functional diversity (FD) and phylogenetic diversity (PD) levels were observed in rivers, lakes and ponds in winter. There were no significant differences in bird diversity within habitats across different seasons, but the species richness (SR) and abundance of ecotypes were significantly different amongst the four seasons, indicating notable seasonal fluctuations in the species composition of the community in this area. Aquatic habitats such as the lakes, rivers and ponds are the most abundant areas of bird diversity in the wetland complex in the Huaibei Plain, providing stop-overs and wintering places for migrants on the East Asian-Australasian Flyway. Meanwhile, the autumn and winter are the key periods for waterbird protection in this area. Our study provides important baseline data on the spatial and temporal distribution patterns of bird communities in the Huaibei Plain and may help in developing scientifically effective management and conservation plans.

## Introduction

Wetlands are one of the world's most productive ecosystems, with rich biodiversity and habitats for many endangered species (Getzner 2002, Ntongani and Andrew 2013). Wetland biodiversity not only contributes to the stability of ecosystems, but also plays an important role in sustainable human development (Garcia et al. 2021). Wetlands with significant habitat heterogeneity have abundant bird communities, often including rare and endemic species (Rajpar and Zakaria 2011, Mereta et al. 2021). Many studies have shown that there are significant differences in bird communities in different types of wetlands such as rivers, lakes, ponds, paddy fields and swamp forests (Derebe et al. 2023, He et al. 2023, Prajapati et al. 2023). There are other studies that show the composition and diversity of wetland bird communities vary significantly in different seasons due to the influence of bird migration and water level fluctuation (Ferrarini et al. 2023).

The above studies mainly focused on the analysis of community species composition, diversity and evenness, usually using traditional diversity indicators such as species richness and taxonomic diversity. However, species richness is susceptible to sampling intensity and density of individuals (Gotelli and Colwell 2001). In light of this, Clarke and Warwick proposed a taxonomic diversity method, comprehensively assessing diversity levels by calculating the average taxonomic distinctness index and the variation in taxonomic distinctness index (Warwick and Clarke 2003). Despite the advantages of this method, its limitations are obvious, such as the inability to reflect the historical status of species, genealogical relationships and the ecological functions of different species in community construction. Compared to traditional diversity metrics, functional diversity (FD), based on Niche Theory and the Limiting Similarity Principle, can more effectively explain ecosystem functioning (Vandermeer 1972; Abrams 1983; Cadotte et al. 2011). According to these theories, species achieve niche separation through the differentiation of functional traits, thereby reducing competition and promoting co-existence. High dissimilarity between species leads to greater Functional Diversity (FD), thereby providing an index of niche complementarity and the diversity of ecological interactions within communities (Cadotte et al. 2011, Chapman et al. 2018). Research indicates that Functional diversity (FD) is a strong predictor of ecosystem productivity and vulnerability (Schleuter et al. 2010). The stability and efficiency of ecosystems are expected to be higher when more multifunctional features are present (Cardinale et al. 2012). Phylogenetic diversity (PD) is based on the theories of phylogenetic signal and Darwin's niche conservatism (Wiens and Graham 2005), characterises the patterns of genetic variation of species in a community and evolutionary relationships amongst species in a community (Muvengwi et al. 2022). Faith defines phylogenetic diversity (PD) as the total phylogenetic distance between two or more species, explicitly measuring differences between species rather than the number of species or their traits (Faith 1992). Phylogenetic diversity (PD) is inversely related to the evolutionary relatedness of species; the more distantly related the species are, the higher the PD (Venail et al. 2015). Higher phylogenetic diversity means that more evolutionary history is preserved (Frishkoff et al. 2014, Jetz et al. 2014). By conserving phylogenetic diversity, the likelihood of losing unique ecological and phenotypic traits within a community can be reduced (Matos et al. 2016).

The Huaibei Plain is located in the south of the Huang-Huai-Hai Plain in eastern China, with flat terrain and mainly used for planting dryland crops. The Huaihe River and its tributaries, such as the Ying River and the Hong River, flow through the Huaibei Plain, providing the region with abundant water resources. In addition, the Huaibei Plain has a variety of habitat types, including farmland, ponds, lakes, woodlands etc., providing foraging, breeding and overwintering sites for birds, particularly the long-distance migrants along the East Asian-Australasian Flyway. However, there are few studies on the diversity of birds in important wetlands in Huaibei Plain (Yongmin et al. 2017). In this context, we aimed to assess 10 provincially significant wetlands in the Huaibei Plain dynamics using multi-dimensional diversity indicators to analyse different aspects of bird diversity in space (habitats) and time (seasons). Our results may reveal the community structure, spatial and temporal distribution patterns and habitat selection preferences of birds in the wetlands in the Huaibei Plain and provide reference suggestions for biodiversity conservation and ecological restoration.

## Methods

**Study Area**

The Huaibei Plain (114°50′-118°18′E, 32°39′-34°44′N) is located in eastern China. The region experiences a warm temperate, semi-humid monsoon climate, characterised by distinct seasons. The region has an average annual temperature of approximately 16.9°C and an average annual precipitation of 884.7 mm.

**Figure 1.**
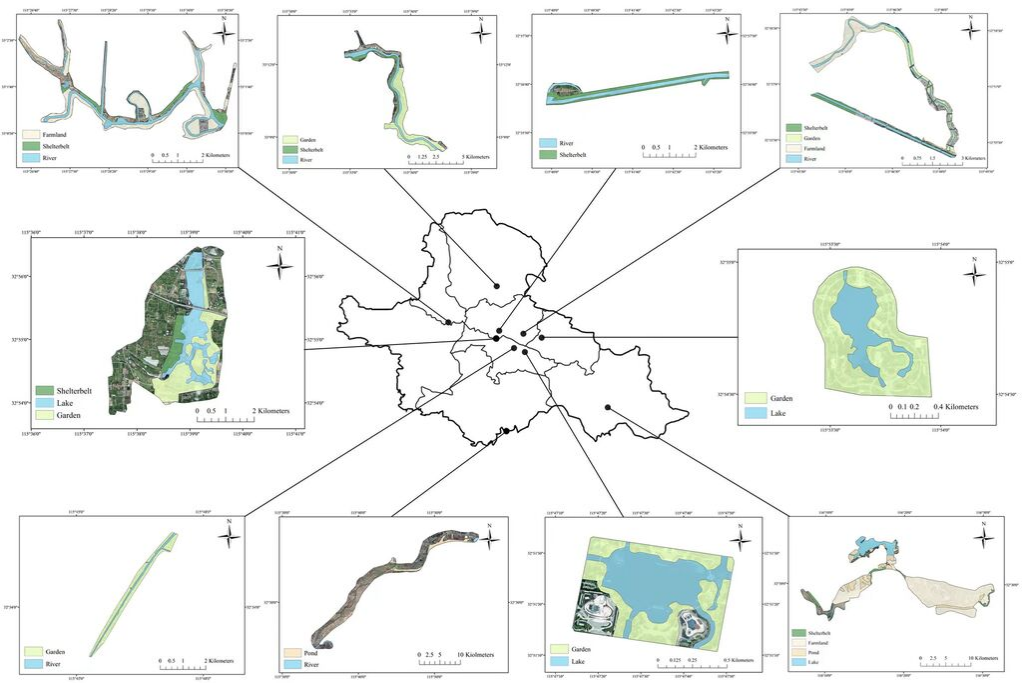
The location and general situation of 10 provincially significant wetlands in Huaibei Plain.

This study selected 10 provincially significant wetlands in the Huaibei Plain, namely the Balihe Provincial Nature Reserve (BPNR), the Wangjiaba National Wetland Park (WNWP), the Yingzhou West Lake National Wetland Park (YWWP), the Liangwan National Wetland Park (LWWP), the Yingzhou West Lake Provincial Nature Reserve (YWNR), the Shayinghe National Wetland Park (SNWP), the Quanshuiwan National Wetland Park (QNWP), the Shuangqingwan Wetland Park (SWP), the Yuejiahu Wetland Park (YWP) and the Qiyuhe Wetland Park (QWP) (Fig. 1) .

**Bird Survey**

From March 2022 to February 2023, monthly bird surveys were conducted using transect and point-count methods across 10 provincially significant wetlands and their surrounding areas. Based on the wetland area, habitat complexity and accessibility of the wetland complex, sampling lines and points were established following the principle of randomness in conjunction with the specific conditions of different habitats. This included 11 sample lines in river habitats, 17 sample points in lake habitats, three sample points and three sampling lines in pond habitats, 10 sample lines in shelterbelt habitats, seven sample lines in garden habitats and four sample lines in farmland. Surveys were conducted during peak bird activity hours (06:00-09:00 h in the morning) under clear, windless weather conditions. The investigator advanced along the line transect at a speed of 1-2 km/h and recorded the birds that were visually observed or heard within 50 m to the front and on both sides of the line transect. To avoid errors caused by duplicate data recording, birds flying from behind the observers towards the front were not recorded. The point count method was applied to large waterbodies, with observation points strategically placed to cover the visible water area and record all birds within the field of view of each sample point. The direct counting method is used when the number of birds is small and the group counting method is used for larger groups of waterbirds. The survey tools included 10 × 42 binoculars (Olympus 10 × 42 PRO) and 60 × 80 monocular telescopes (Celestron C20-60 x 80A) and a portable GPS device to determine the location, length and orientation of the transects.

**Statistical Analyses**

The differences in seasons and habitats on the multi-dimensional diversity of birds were analysed using two-way ANOVA, which was also used to analyse the ecotype differences amongst bird species. Significant tests were further subjected to a Tukey's honestly significant difference test when p-value was < 0.05. All analyses were performed with R version 4.4.1.

**Species Diversity Analysis**

Species diversity reflects the stability of community structure. In this study, species richness, Shannon-Wiener index (H') and Pielou index (E) were used to describe bird species diversity (Shannon 1950, Pielou 1966). The formulae are as follows:

Species Richness: Number of bird species in a single survey.

Shannon-Wiener index (H'):

\begin{varwidth}{50in}\begin{equation*}
            H^{\prime}=-\sum_{i=1}^{S}\left(P_{i}\right)\left(\ln P_{i}\right)
        \end{equation*}\end{varwidth}

Pielou index (E):

\begin{varwidth}{50in}\begin{equation*}
            E=H'/lnS
        \end{equation*}\end{varwidth} ,

where *S* is the total number of bird species, Pi is the proportion of the individual number of the *i*-th species to the total number of individuals in the community.

Taxonomic DiversityTaxonomic diversity (TD) is used to reflect the taxonomic composition of bird communities (Warwick and Clarke 2003). The formula is as follows:

\begin{varwidth}{50in}\begin{equation*}
            \boldsymbol{T D}=\frac{\sum \sum_{i j} \omega_{i<j} X_{i} X_{j}}{\sum \sum_{i<j} X_{i} X_{j}+\sum_{i} X_{i}\left(X_{i}-1\right) / 2}
        \end{equation*}\end{varwidth} ,

where *dij* is the distance between species *i* and *j*, *Xi* and *Xj* are the abundance of species *i* and species *j*.

Functional diversity (FD) reflects the response of biological communities to ecosystems. In this study, functional richness (FRic) was selected to quantify functional diversity (Barbaro et al. 2013). One continuous variable (body weight) and four categorical variables (food type, residence type, feeding location and group behaviour) were selected and the functional characteristic data were derived from A Dataset on the Life-history and Ecological Traits of Chinese Birds (Wang et al. 2021). The formula is as follows:

\begin{varwidth}{50in}\begin{equation*}
            FR_{ic} =SF_{ic} /R_{c} 
        \end{equation*}\end{varwidth}

*SFic* refers to the niche occupied by all species in the community and *Rc* refers to the absolute value of the eigenvalue.

Phylogenetic diversity (PD) reflects the uniqueness of bird communities and phylogenetic relationships. Based on species survey data, evolutionary information for the species was obtained from the Timetree of Life (https://timetree.org/home) (Kumar et al. 2022). The R package *picante* was used to calculate Faith's phylogenetic diversity, which represents the total branch length of the minimum subtree encompassing all species on the phylogenetic tree (Sandel 2017). The formula is as follows:

\begin{varwidth}{50in}\begin{equation*}
            PD=\sum c_{i} l_{i} 
        \end{equation*}\end{varwidth} ,

Where *ci* is the number of the *i* ^th^ species and *li* is the length of independent branches of that species on the phylogenetic tree.

## Results

**Bird community**

**Figure 2.**
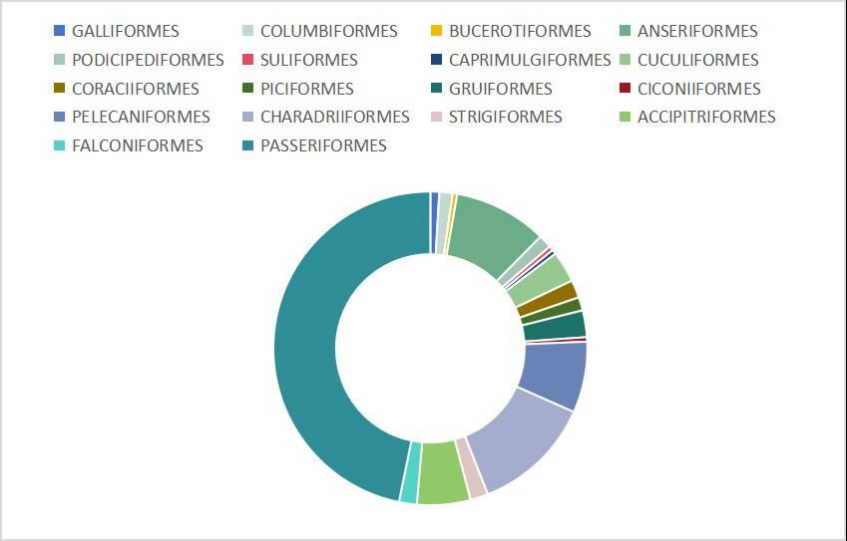
Number of species recorded from different orders in percent.

**Figure 3.**
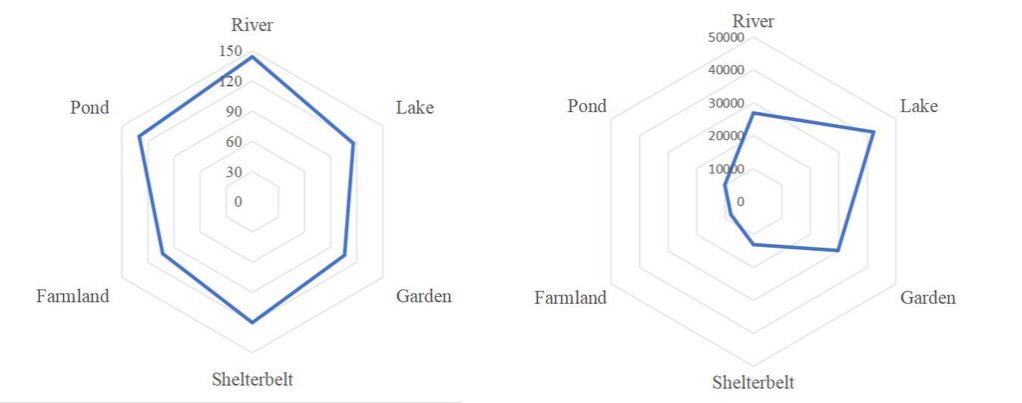
Number of bird species and individuals in different habitats.

During the study period, a total of 129915 individual birds, including 217 species, 18 orders and 54 families, were recorded in the study area（Suppl. material 1). The order Passeriformer had the highest number of species amongst the recognised species with 47% of the total number of species, followed by Charadriiformes at 12%. Furthermore, Anseriformes (10%), Pelecaniformes (7%) and Accipitriformes (6%) were the third, fourth and fifth most frequently observed, respectively (Fig. 2.) There were differences in bird species and individual numbers across different habitats (Fig. 3).

**Figure 4.**
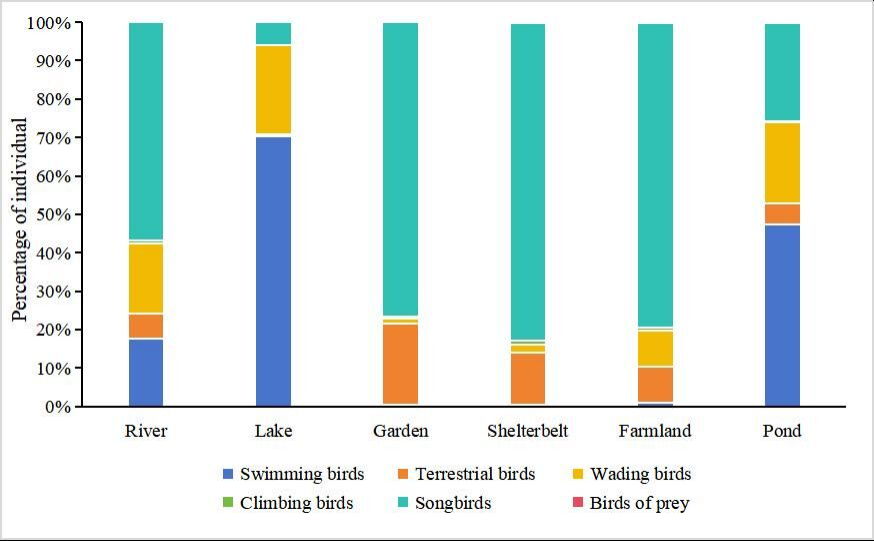
Percentage of individuals with bird community structure in different habitats.

Songbirds predominantly occupied river, garden, shelterbelt and farmland habitats, whereas swimming birds were mainly found in lake and pond habitats, as shown in Fig. 4. Analysis of variance (Table 1) demonstrated that different seasons significantly influenced the abundance and species richness of birds across various ecotypes.

Fifteen species of threatened birds were observed, including the Critically Endangered Baer's Pochard (*Aythyabaeri*), the endangered Oriental Stork (*Ciconiaboyciana*), Lesser Sand Plover (*Charadriusmongolus*), the vulnerable Lesser White-fronted Goose (*Ansererythropus*), Long-tailed Duck (*Clangulahyemalis*), Common Pochard (*Aythyaferina*), Horned Grebe (*Podicepsauritus*), White-naped Crane (*Grusvipio*), Black-capped Kingfisher (*Halcyonpileata*), Rustic Bunting (*Emberizarustica*), the near-endangered Ferruginous Duck (*Aythyanyroca*), Falcated Duck (*Marecafalcata*), Northern Lapwing (*Vanellusvanellus*), Reed Parrotbill (*Paradoxornisheudei*) and Japanese Waxwing (*Bombycillajaponica*), all of which were included in the Red List from the International Union for the Conservation of Nature.

**Bird community characteristics of different feeding guilds**

**Figure 5.**
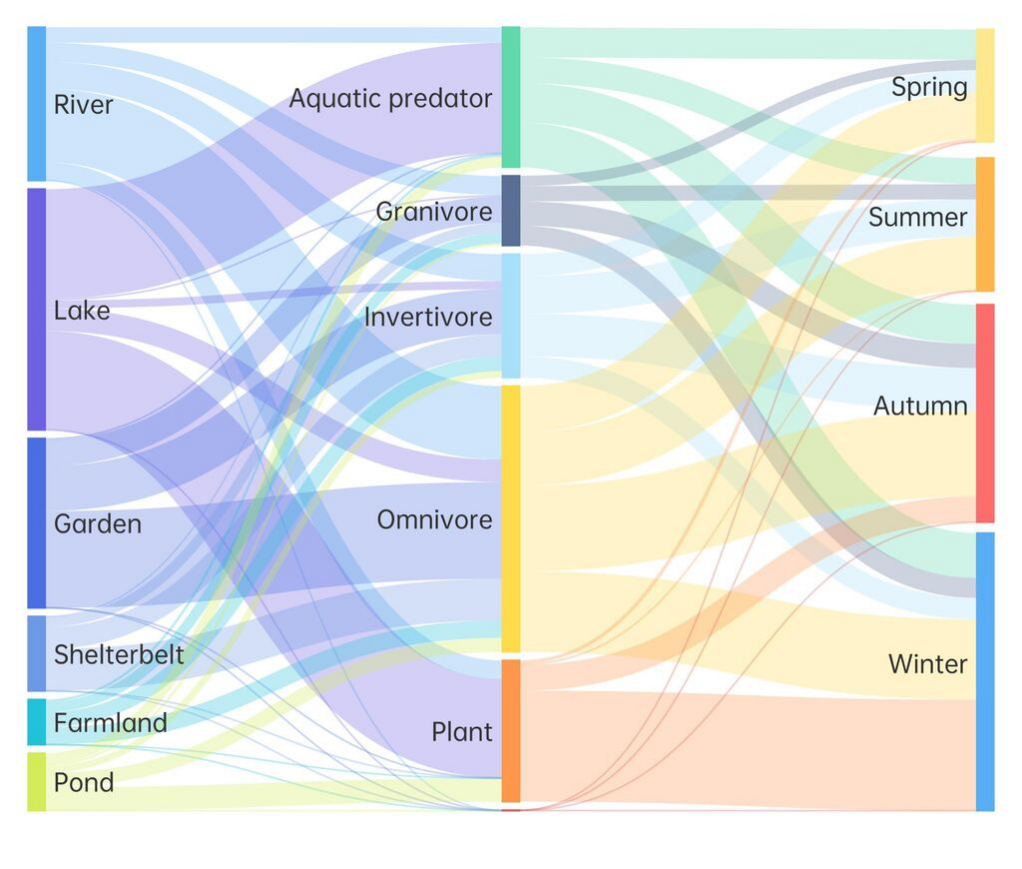
Feeding guild composition characteristics, based on bird individual numbers in different habitats.

As far as different bird feeding guilds are concerned, river habitats were predominantly occupied by omnivorous species. Lake habitats were mainly inhabited by aquatic predators and plants; gardens, shelterbelts and farmlands were primarily dominated by omnivorous and invertebrate species. Ponds were chiefly inhabited by plants. In terms of different seasons, the number of individual birds was more evenly distributed amongst feeding guilds, although the distribution varied in winter (Fig. 5).

**Bird diversity differences within seasons amongst habitats**

**Figure 6.**
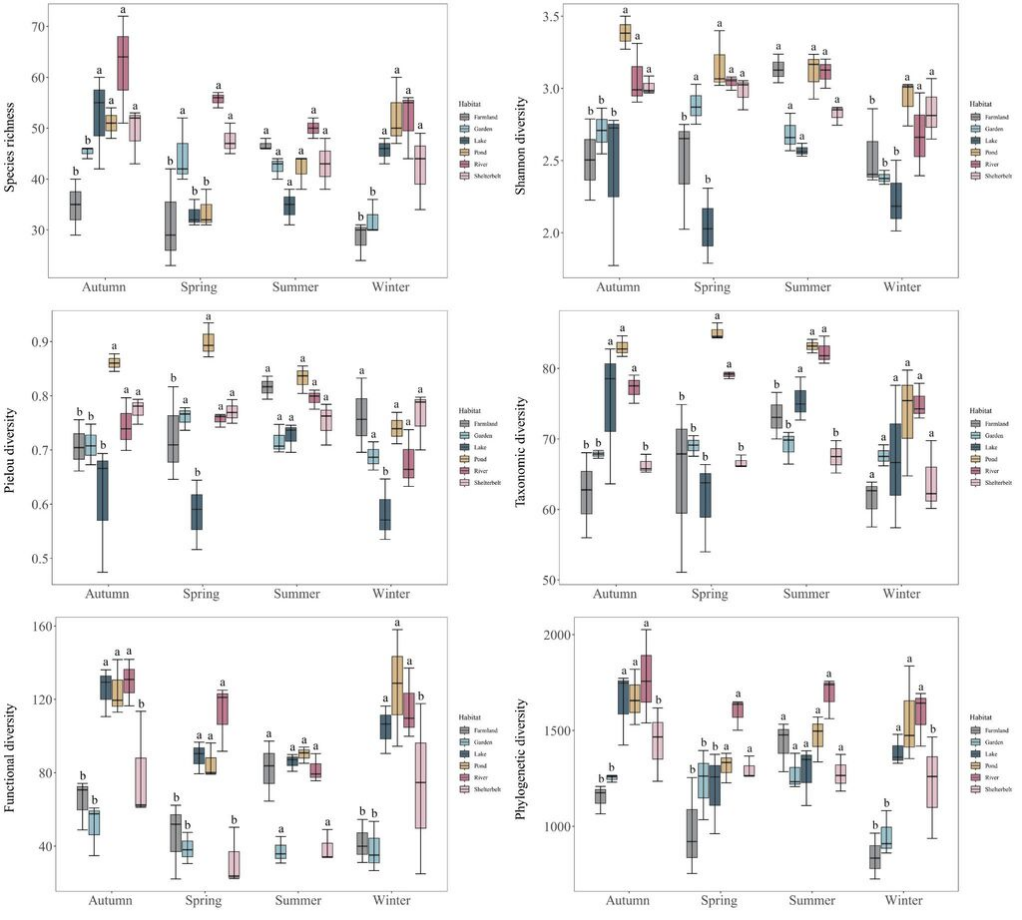
Seasonal differences in multiple biodiversity metrics of bird communities across habitats. Boxplots with the same letters indicate no significant differences.

In spring, garden, shelterbelt and river habitats showed the highest species richness (SR). Ponds and rivers exhibited greater taxonomic diversity (TD) and phylogenetic diversity (PD) indices than other habitats. In summer, farmlands displayed higher species richness (46.67 ± 7.00), Shannon-Wiener (3.13 ± 0.10), Pielou (0.82 ± 0.02) and phylogenetic diversity (1431.31 ± 129.92) indices compared to shelterbelts, gardens and lakes, although these differences were not statistically significant. In autumn, river, lake and pond habitats exhibited markedly higher levels of taxonomic diversity (TD), functional diversity (FD) and phylogenetic diversity (PD) relative to other habitats. Shelterbelt habitats demonstrated notably higher species richness (49.33 ± 11.53), Shannon-Wiener (3.01 ± 0.06) and Pielou (0.77 ± 0.02) indices than farmland and garden habitats (*p < 0.05*). In winter, ponds, rivers and lakes showed clear advantages in functional diversity (FD) and phylogenetic diversity (PD) indices (*p < 0.05*). Amongst all these habitats, gardens and farmlands had the lowest species richness (SR) (Fig. 6).

**Bird diversity differences winthin habitats amongst seasons**

**Figure 7.**
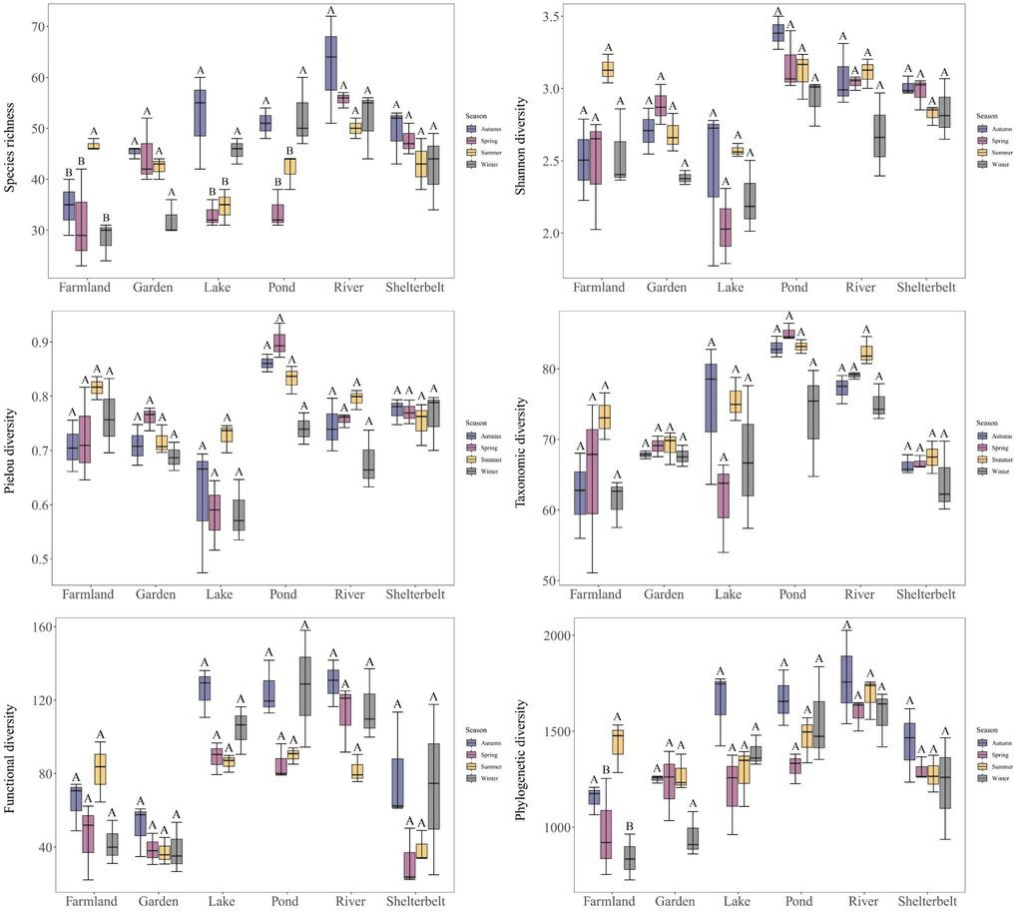
Habitat differences in bird community diversity indices across different seasons. Box plots with the same letters indicate no significant differences.

In river habitats, species richness (62.33 ± 8.50), functional diversity (129.70 ± 12.70) and phylogenetic diversity (1773.91 ± 243.80) were higher in autumn than in other seasons. In lakes, species richness (SR), Shannon-Wiener, taxonomic diversity (TD) and phylogenetic diversity (PD) indices were highest in autumn and lowest in spring, with the seasonal variation in species richness (SR) being statistically significant (*p < 0.05*). In pond habitats, the species richness (SR) index followed the pattern: autumn < winter < summer < spring, with marked differences between seasons (*p < 0.05*). In farmlands, summer exhibits peak values for all diversity indices, with significant seasonal variations in species richness (SR) and phylogenetic diversity (PD) indices. In garden habitats, the seasonal variation pattern for the Shannon-Wiener, Pielou and phylogenetic diversity (PD) indices is as follows: spring < autumn < summer < winter. In shelterbelts, both species richness (49.33 ± 11.53) and phylogenetic diversity index (1439.43 ± 192.42) peaked in autumn, whereas the functional diversity index (32.08 ± 15.77) was lowest in spring, though the differences across seasons were not statistically significant (Fig. 7).

## Discussion

**Spatiotemporal variation in bird diversity**

River habitats exhibit relatively high multidimensional diversity of birds in the four seasons. In spring, the budding leaves of trees and shrubs along the riverbanks attract birds that feed on these tender leaves and insects, making it an ideal breeding season habitat. During summer, emergent plant such as Reeds (*Phragmitesaustralis*) and Giant Reed (*Miscanthussacchariflorus*) form aquatic vegetation communities that support a large number of insects and other small invertebrates, attracting various birds that prey on these organisms. Swimming birds such as Little Grebe (*Tachybaptusruficollis*), Great Crested Grebe (*Podicepscristatus*) and Eastern Spot-billed Duck (*Anaszonorhyncha*), wading birds such as Common Moorhen (*Gallinulachloropus*) and Black-crowned Night Heron (*Nycticoraxnycticorax*) and songbirds such as Oriental Reed Warbler (*Acrocephalusorientalis*) and Black-browed Reed Warbler (*Acrocephalusbistrigiceps*) forage and breed in the river wetlands. Shallow and muddy areas in the river provided rich benthic animals and small fish as food sources for gulls and herons, further enhancing bird diversity. In autumn, aquatic plants such as lotus roots (*Nelumbonucifera*) and water chestnuts (*Trapabispinosa*) gradually mature and bear fruit and the number of fish and aquatic insects is at its peak, making the rivers important energy replenishment sites and stop-overs for migratory birds. In winter, riverside shrubs and reeds provide foraging sites and shelters for small passerine birds (e.g. *Sinosuthorawebbiana*, *Emberizaspodocephala*, *Passermontanus* etc.), which makes the river habitat maintain the high bird diversity (Verma and IFS 2023).

In lake wetlands, the functional diversity (FD) and phylogenetic diversity (PD) are relatively high in the autumn and winter. In early autumn, some summer migrants are still recorded in the lakes, while in late autumn, winter migrants such as Scyther Duck (*Anasfalcata*) and Ruddy Duck (*Anasstrepera*) have already arrived, forming a complex bird community. In winter, the wide open water surface, abundant fish resources and submerged plants in the lake wetlands provide a foraging place for large flocks of overwintering birds such as geese, ducks and cormorants. The islands in the lake provide a place for these birds to stay overnight, making the lake wetlands important wintering places for ducks in the East Asian-Australasian Flyway (La Sorte et al. 2022).

The ponds feature high levels of diversity across functional, taxonomic and phylogenetic aspects. In spring, as the temperature rises, the plants in the pond begin to germinate and the number of insects increases significantly, providing ample food resources for birds such as migrating plovers, further improving species richness and evenness. In summer, the proliferation of aquatic organisms such as fish, shrimp and plankton within the pond attracts various waterbirds, including gulls and herons, to forage. Additionally, the leaves of euryale ferox provide ideal breeding grounds for species like the Common Moorhen (*Gallinulachloropus*) and Pheasant-tailed Jacana (*Hydrophasianuschirurgus*). In autumn, as water levels recede, more shallow areas and benthic organisms are exposed, providing abundant foraging opportunities for shorebirds. During winter, despite the reduced activity of aquatic organisms, submerged lotus rhizomes and the remaining fish and shrimp in lotus ponds and fishponds continue to attract waterbirds such as swans, herons and ducks for foraging and overwintering (Hsu et al. 2019). Moreover, the relative stability of the pond ecosystem provides a long-term and reliable habitat for the birds, which enables the bird populations to continue to reproduce, thus further enhancing the diversity of pond birds.

Gardens in the spring show high levels of species diversity, probably due to the combination of tree species, shrubs and grass, which directly or indirectly attract and support roosting and breeding of granivorous and insectivorous birds (Li et al. 2019). In farmland habitats, bird diversity peaks in the summer. Insects and aquatic animals in irrigation canals serve as food sources for wading birds such as the Chinese Pond-Heron (*Ardeolabacchus*) and Black-crowned Night Heron (*Nycticoraxnycticorax*). In terms of seeds and fruits produced by various crops and weeds, ample foraging resources are provided for birds such as the Oriental Magpie (*Picaserica*), Oriental Turtle-Dove (*Streptopeliaorientalis*) and Eurasian Tree Sparrow (*Passermontanus*). Shelterbelt habitats have higher levels of species diversity in autumn and winter. In autumn, shelterbelts with heterogeneous vegetation and diverse layers provide several niches for birds, making these areas habitats with high bird diversity, the outcome of this result coinciding with the report of de Zwaan (de Zwaan et al. 2024). Moreover, the temporary aggregation of migratory birds further enhances bird species diversity and the shelterbelts also provide nocturnal roosts for some forest birds in autumn and winter.

**The important role of the Huaibei Plain wetland complex in the East Asian-Australasian Flyway**

The Huaibei Plain wetland complex is adjacent to the north bank of the Huaihe River and has a well-developed water system. It provides important stop-over sites and suitable overwintering habitats for birds travelling along the East Asian-Australasian flyway. In this survey, 31 species of swimming birds were recorded, with a total of 39,567 birds. Amongst them, the Great Cormorant (*Phalacrocoraxcarbo*) and the Common Pochard (*Aythyaferina*) were more than 3000 in a single survey in the Balihe Provincial Nature Reserve. In addition, there were 15 Baer's Pochard (*Aythyabaeri*) in a single survey in the Yingzhou West Lake National Wetland Park. The number of individuals of these three species in a single survey exceeds 1% of the total population in the East Asian-Australasian flyway (http://wpe.wetlands.org). According to the Ramsar convention on wetlands, the Yingzhou West Lake and Balihe meet the wetlands of international importance standards, indicating that the Huaibei Plain wetland complex is an important part of the international wetland network.

## Conclusion

These lakes, rivers and ponds are the richest areas in terms of bird diversity in this area; furthermore, autumn and winter are the important periods for waterbird protection. Farmlands and woodlands surrounding the aquatic habitats provide varied food sources, breeding sites and nocturnal resting sites for wetland birds and are an important part of the wetland complex. The wetland complex in the Huaibei Plain could provide critical habitats for a wide range of bird species, particularly the long-distance migrants along the East Asian-Australasian Flyway. Therefore, strengthening the protection and management of these wetlands is crucial for maintaining biodiversity and wetland functions. In terms of different habitats, river habitats require conservation of shrubs/mudflats during the breeding season and reed communities during migration periods. Lake habitats should maintain open water areas, have reduced human disturbances on mid-lake islands and provide nesting and roosting sites for waterbirds. Pond habitats should establish a gradient structure of "deep water- shallow beach-muddy land", retain floating-leaved vegetation in summer to support breeding waterbirds and adopt rotational harvesting in lotus ponds to ensure winter food supply for migratory birds.

## Supplementary Material

628F4698-784D-57C2-9BD2-EB3EBC489A5F 10.3897/BDJ.13.e154264.suppl1 Supplementary material 1 Bird data of 10 provincially significant wetlands in the Huaibei Plain Data type xlsx File: oo_1293000.xlsx https://binary.pensoft.net/file/1293000 Yongmin Li

## Figures and Tables

**Table 1.** Effects of four seasons on species richness and abundance of different bird ecotypes.

	Spring		Summer		Autumn		Winter	
	Richness	Abundance	Richness	Abundance	Richness	Abundance	Richness	Abundance
Swimming birds	8.00±2.65^c^	1016.33±1276.27^bc^	5.33±0.58^dc^	509.33±177.43^dc^	14.00±2.65^bc^	3048.33±2035.28^b^	22.00±2.00^b^	8615.00±3083.39^a^
Terrestrial birds	6.00±0.00^c^	563.00±49.57^bc^	5.67±0.58^dc^	928.00±89.27^c^	5.33±0.58^c^	1309.33±218.51^c^	5.33±0.58^d^	941.33±95.14^b^
Wading birds	15.33±2.52^b^	1640.00±112.93^b^	21.00±3.61^b^	1684.00±330.61^b^	21.33±6.11^b^	1576.33±200.15^c^	14.33±3.21^c^	1180.33±104.52^b^
Climbing birds	7.67±1.15^c^	51.67±40.28^c^	9.67±0.58^c^	90.33±34.36^d^	4.00±0.00^c^	34.67±5.69^c^	5.00±1.00^d^	32.33±5.86^b^
Songbirds	56.00±1.73^a^	3297.33±252.94^a^	39.33±2.52^a^	4567.67±346.37^a^	58.67±6.66^a^	6733.33±392.00^a^	43.00±3.61^a^	5454.33±872.22^a^
Birds of prey	4.33±0.58^c^	6.00±1.00^c^	2.00±2.00^d^	2.33±2.08^d^	9.33±0.58^c^	17.67±3.21^c^	4.00±1.00^d^	6.33±3.21^b^
